# Homeless-experienced patients’ perspectives on improving patient portal Use

**DOI:** 10.3389/fdgth.2026.1861993

**Published:** 2026-09-08

**Authors:** Mikayla O. Castellon, Talia J. Panadero, Elizabeth M. Moore, Sonya E. Gabrielian

**Affiliations:** 1 Department of Veterans Affairs Greater Los Angeles, VA Center for the Study of Healthcare Innovation Implementation and Policy (CSHIP), Los Angeles, CA, United States; 2 Department of Veterans Affairs Greater Los Angeles, Veterans Integrated Services Network 22 Mental Illness Research, Education, and Clinical Center (MIRECC), Los Angeles CA, United States; 3 Department of Psychology, Undergraduate Research Scholarship Program (URSP), University of California Los Angeles, Los Angeles, CA, United States; 4 Department of Psychiatry, University of Pennsylvania Perelman School of Medicine, Philadelphia, PA, United States; 5 Department of Psychiatry and Biobehavioral Sciences, University of California Los Angeles David Geffen School of Medicine, Los Angeles, CA, United States

**Keywords:** digital health, health equity, homelessness, housing, implementation science, patient portal, permanent supportive housing, veterans

## Abstract

**Background:**

Persons who have experienced homelessness (PEHs) face profound health disparities and barriers to digital health access. Among Department of Veterans Affairs (VA) patients experiencing homelessness, only 36% (vs. 70% of all VA users) are enrolled in the VA's digital patient portal: My HealtheVet (MHV). MHV allows patients to access health information and communicate with providers.

**Objective:**

We conducted an exploratory qualitative study to identify PEHs’ perspectives on MHV engagement and inform equity-focused strategies to support portal adoption.

**Methods:**

We conducted semi-structured interviews with 17 PEHs at VA Greater Los Angeles who enrolled in MHV and participated in the Department of Housing and Urban Development-VA Supportive Housing (HUD-VASH) program, which combines housing subsidies with case management for Veterans experiencing homelessness. Interviews explored MHV enrollment, uses, barriers and facilitators to engagement, and recommendations to improve adoption. Data were analyzed using rapid qualitative methods.

**Results:**

PEHs valued MHV for secure messaging, medication management, and medical record access. Technical challenges, including login difficulties, hindered engagement. Housing stability in HUD-VASH facilitated MHV use via reliable technology, whereas homelessness itself was a barrier. Peer networks supported MHV adoption through knowledge exchange. Participants recommended simplifying interfaces, integrating MHV training into HUD-VASH intake, increasing technical support from VA staff, and providing devices to address gaps.

**Conclusions:**

MHV utilization may improve health engagement among PEHs. However, its benefits are limited by persistent digital inequities. Targeted strategies—such as peer-led onboarding, interface simplification, and integration of MHV support within housing services—may improve uptake and promote equitable access to digital healthcare.

## Introduction

Persons who have experienced homelessness (PEHs) face profound health inequities compared to their housed peers, including a life expectancy of 42–52 years compared to a national average of 78.6 years (1). These disparities are exacerbated by fragmented healthcare utilization, with high rates of acute care use and low rates of primary care and preventive services, which contribute to PEHs’ poor long-term health outcomes (2). Digital health technologies, including patient portals—web-based tools that enable patients to access and manage their health information and communicate with providers—are important tools to address these disparities. Yet, we know little about how patient portals are used and experienced by PEHs.

The Department of Veterans Affairs (VA) has prioritized ending Veteran homelessness and widely disseminated robust health and social services for PEHs (3, 4). PEHs enrolled in VA healthcare can access health and social services in VA's national integrated system, including services specifically tailored for PEHs. These services include VA's digital health technologies, such as My HealtheVet (MHV), the VA's patient portal that was launched in 2003. MHV enables remote prescription refills, appointment scheduling, secure provider messaging, and access to personal health records. By 2019, MHV had over 5 million registered users, with accelerated adoption during the 2019 coronavirus (COVID-19) pandemic (5). However, enrollment among VA users who are PEHs remains disproportionately low at only 36% (vs. 70% in the general VA patient population).^†^

Studies reveal substantial mobile phone ownership rates among PEHs, signaling the potential value of digital health interventions for this population (6–8). Disparities in VA portal adoption among PEHs likely reflect broader digital-divide barriers, encompassing limited digital health literacy and mistrust of health care institutions or digital platforms (9, 10). These disparities are likely driven by multilevel factors, including system-level barriers such as fragmented and poorly integrated care systems, as well as patient-level challenges that disproportionately affect people with mental health conditions and experiences of homelessness (11, 12). However, the specific contextual factors that support or impede digital health intervention use among homeless-experienced patients are poorly understood.

To address this gap, we conducted an exploratory qualitative study as part of a larger project examining MHV use among Veterans engaged in the VA's permanent supportive housing program [the Department of Housing and Urban Development-VA Supportive Housing (HUD-VASH) initiative]. HUD-VASH is the core of VA's strategic plan to end Veteran homelessness, combining financial subsidies for permanent and independent housing integrated with field-based supportive services and case management (3, 13, 14). We sought to identify systemic-, organizational-, and individual-level factors affecting MHV adoptions among homeless-experienced Veterans and to inform equity-focused strategies that support patient portal adoption among PEHs within and outside VA. This integrated, supportive model positions HUD-VASH case managers as critical individuals who could potentially facilitate access to and utilization of MHV among PEHs on their caseload.

## Methods

### Setting

The study was conducted at the VA Greater Los Angeles Healthcare System, a large, tertiary care medical system that spans Southern California, including Los Angeles, Ventura, Kern, Santa Barbara, and San Luis Obispo counties. We specifically focused on Veterans enrolled in HUD-VASH, which provides case management and permanent housing subsidies. Through HUD-VASH, there are over 8,000 permanent housing subsidies available within the greater Los Angeles area, and nationally nearly 200,000 Veterans have received services in HUD-VASH since its inception (15). All study activities were approved by the VA Greater Los Angeles Institutional Review Board.

### Study design

This exploratory qualitative study was conducted as part of a larger parent project examining MHV use among Veterans engaged in HUD-VASH. The parent project used VA administrative data to examine clinical factors associated with MHV use. The present study used a brief structured questionnaire and semi-structured qualitative interviews with a purposively selected subset of Veterans from the parent cohort to examine systemic-, organizational, and individual-level factors affected MHV adoption.

### Participant recruitment and consent

For the parent project, a list of (*n* = 11,363) Veterans engaged with HUD-VASH during the 2019 calendar year was abstracted from VA's homeless registry [the Homeless Operations and Management Evaluation System (HOMES) database]. Using administrative data from VA's electronic health record, we identified the sub-cohort aged 18 years or older who were enrolled in MHV as of 2019 (*n* = 4,084). For the present study, we used purposive sampling—seeking diversity in age, gender, and race/ethnicity—to identify 70 participants who were verified MHV users with at least one MHV login and one accessory use of the portal (verified prescription refill, secure message, or viewed medical note) from 2009 to 2019.

Recruitment letters were mailed to these 70 Veterans (20 initially, and then 50 following an initial 9 interviews), including a phone number to opt out or inquire about the study. After a two-week opt-out period, VA staff contacted remaining Veterans twice by phone to seek their voluntary participation in this study. We obtained verbal informed consent and Health Insurance Portability and Accountability Act of 1996 (HIPAA) authorization via Docusign prior to data collection from all participants (*n* = 17).

### Data collection

All data collection was conducted by telephone by the first author (M.C.). Telephone data collection was selected to facilitate participate across the geographically dispersed VA Greater Los Angeles catchment area and reduce transportation and other logistical barriers to in-person participation.

First, participants were administered a brief questionnaire asking about demographics, healthcare engagement, and digital access. Demographics included age, gender, race, ethnicity, marital status, and housing status [i.e., housed, sheltered homelessness [temporary housing, hotel/motel, doubling up], unsheltered homelessness [car, park, street, other place not meant for human habitation], institutional housing [rehabilitation, inpatient program]]. Healthcare engagement items assessed the frequency of medical visits in the past 12 months, whether participants had a regular healthcare provider and routine medication use. Digital access items assessed MHV account knowledge, access to a working cell phone and smartphone, recent text messaging behavior, computer access, and internet use. These items were adapted from previously published measures of telehealth access and digital technology use (16). We ascertained participants’ MHV account type. Any user who registers with MHV is provided with access to self-entered health information via a “Basic” account. Users have the option to upgrade to a “Premium” account to view key information from their VA health records (e.g., discharge summaries, laboratory results). The Premium account also offers access to fully integrated VA and Department of Defense (DoD) medical records (17). The full list of structured questionnaire items is provided in the Supplementary Materials.

Following the questionnaire, we conducted a semi-structured interview (∼30 min/each) in which we queried participants about ways they used MHV, enrollment experiences, perceived benefits/challenges with the platform, the relationship between MHV use and housing status, and recommendations for MHV improvement. The interviews were audio-recorded and transcribed using Microsoft Teams. Each participant received a $30 gift card for completing data collection.

### Data analyses

Interviews were audio-recorded and transcribed using Microsoft Teams’ automated transcription function. Microsoft Teams-generated transcripts were reviewed after each interview and verified against original recordings. We applied rapid qualitative analysis to these transcripts using a structured matrix organized around predefined domains from the semi-structured interview guide, including MHV enrollment, utilization, barriers, facilitators, the influence of housing status, and suggestions for improvement (18, 19). These methods have precedent within a breadth of health services research studies in applied health care systems (20–22). Analyses were primarily deductive, with recurring patterns and findings identified within and across these domains.

Two authors (M.C. and T.P.) conducted the analysis. To establish a shared approach to applying the matrix, both analysts independently summarized the same initial transcript and compared summaries. Differences in interpretation or application of the matrix were discussed until consensus was reached, after which the remaining transcripts were divided between the two analysts for individual summary. Following completion of interview-level summaries, both analysts reviewed the summarized data across all interviews by domain and collaboratively developed a cross-case “summary of summaries.” Recurring findings were identified and refined, and discrepancies in interpretation were resolved through discussion and consensus.

The summary of summaries included key findings, descriptive counts of participants who reported particular experiences or perspectives, and illustrative quotations. Counts were used to characterize how commonly specific findings arose within the sample. The summary of summaries, resulting findings, and illustrative quotes were reviewed and discussed with the full authorship team and formed the basis for the themes presented in the Results (23).

## Results

### Sample

Participants (*n* = 17 PEHs) had a mean age of 53.8 ± 10.8 years. All participants had previously been or were currently housed through HUD-VASH and were mostly Non-Hispanic/Non-Latino (82%). About half identified as Black or African American (47%), followed by White (41%). More than half of the participants were Never Married (53%) and about a third were Divorced or Separated (35%).

### Healthcare and digital access

Most participants (94%) reported having a regular doctor or physician and all participants reported taking routine medications.

All participants reported having a working smartphone and utilizing it at least once in the past 30 days to send or receive text messages. Most participants (82%) reported access to a computer and all participants reported accessing the internet.

### My HealtheVet use

The majority (82%) of participants utilized My HealtheVet (MHV) premium accounts, requiring identity verification and alignment of profile data (name, SSN, date of birth, sex) with VA/DoD for full feature access, while only one retained a basic account limited to self-entered health tracking without VA/DoD integration (24). Participants utilized MHV in a variety of ways, including sending secure messages to their care team (94%), tracking prescriptions (88%), medication refill requests (77%), viewing medical notes (77%), scheduling appointments (59%), and utilizing health education resources (12%).

### Interview findings

Salient findings are summarized in Table 1 and detailed below. Participants highlighted MHV's utility in managing their healthcare, key technological and housing-related barriers to sustained engagement with MHV, and opportunities (via peer networks and systemic improvements) to improve equity in portal adoption.

**Table 1:** Key findings and exemplar quotations.

Key Finding	Exemplar Quotation
1. MHV Enhances Healthcare Access and Autonomy	*“I don't have to wait weeks to see my primary care if I have questions” and “I can see my prescriptions when I want, I can leave messages in the middle of the night if I want.”*
2. Technological Barriers Hinder Portal Engagement	*“[My doctors’ visit notes] are not updated– there will be a prompt saying, ‘we are updating’ and it never gets updated.”*
3. Housing Stability Facilitates MHV Utilization	*“Homelessness comes with its own challenges, but one of them is not having internet or WIFI usage when you need it”*
4. Peer Networks Facilitate Portal Adoption	*“I just help people [..] and I introduce my friends to MHV [..] because nobody did it for me until 40 years later”*
5. Veterans Suggest the Value of Systemic Reforms	*“You [referring to VA case managers] finally made contact with a Veteran who needs help with the housing [..] You should show them how to sign up [for MHV]. Show them how to navigate [MHV] and then make it to where you don't have to change that password every 30 days”*

#### MHV enhances healthcare access and autonomy

PEHs widely utilized MHV to enhance their healthcare access and increase their autonomy regarding their healthcare needs. Many valued the portal for streamlining communication, noting that providers “get back to you really quick.” Several Veterans frequently utilized MHV to manage their chronic conditions and medical problems by reviewing lab results and comparing them to previous ones, with one participant describing the utility of “even looking at the x-rays”. The ability to share health data with non-VA providers was important to participants; as one Veteran explained, “I can give [non-VA providers] access because I have immediate access and it's a digital copy”. For those managing chronic illnesses like Type 2 diabetes, health education tools provided “healthy tips for eating,” and the appointment scheduling feature was utilized primarily for independent care management among PEHs with chronic conditions. Daily or weekly engagement was common among Veterans managing complex illnesses. Others relied on biweekly or monthly use for essential tasks like prescription refills, reflecting varied but vital healthcare needs.

#### Technological barriers hinder portal engagement

Participants described several barriers to using MHV, including login difficulties, authentication delays, technical errors, and frequent password resets during account migration. Nearly half of participants encountered login difficulties, especially with the changes in 2025 to VA's secure identity verification platforms [from Defense Self-Service Logon (DS Logon) to ID.me] (25). Dual verification login requirements (i.e., ID.me) were a common complaint; one participant stated, “that extra step doesn't make any sense.” Multiple participants criticized the portal's design, describing it as “counterintuitive” and difficult to navigate when attempting to reach human support online or locating appointment features. Others pointed to age-related challenges, suggesting that the platform needs to be “a little bit more computer friendly for the older people”. A few participants also requested digital literacy classes. Technical support gaps were found, with participants reporting unresponsive technical assistance, with one stating, “No one should have to leave voice messages during business hours”. Notably, few actively sought support with the MHV from HUD-VASH case managers, primary care team members, or other VA staff, instead relying on technological support seemingly dedicated to the MHV program. These frustrations were compounded for vulnerable groups, including those self-reported post-traumatic stress disorder (PTSD), cognitive decline, or limited digital literacy.

#### Housing stability facilitates MHV utilization

Participants described stable housing through HUD-VASH as supporting MHV use, by increasing access to internet services, devices, and caseworker assistance. One participant believed MHV “would be a big benefit if more people were to use it, especially in HUD-VASH.” In contrast, participants described periods of homelessness as making portal use more difficult, particularly when internet access was limited, or when immediate needs such as shelter took priority. One Veteran emphasized that “trying to figure out where [they’re] gonna stay” outweighed the desire to access healthcare remotely via MHV. Post-housing stability increased reliance on MHV for managing VA benefits and documents. However, persistent connectivity issues remained a barrier even for housed Veterans, with one noting, “sometimes the internet fails me.”

#### Peer networks facilitate portal adoption

Peer networks­—via informal connections with fellow Veterans and interactions with VA peer-support staff—were described as an important sources of information about MHV. Some reported learning about the portal through fellow veterans or VA peer-support staff. One Veteran noted: “Everyone's got a little bit of information.” Some participants described relying on Veteran peer networks for information about MHV, particularly when formal outreach was limited or when they did not receive information from care teams. Some self-taught users became advocates for MHV, with one stating, “I do a lot of research on MHV” to help others. As one Veteran pointed out, “The average Veteran doesn't have [information about MHV], and when you go in and talk to your primary care, they don't bring it up”.

#### Recommendations for MHV improvements

Many participants suggested ways to improve MHV access and use. They suggested that VA staff and providers (e.g., caseworkers) “be more patient and willing to try and assist” with MHV issues. Others called for VA-funded device distribution, with one noting, “having the VA provide a phone [..] would be helpful”. One Veteran recommended that the VA “make being part of MHV a part of HUD-VASH.” Additional ideas included more vigorous public outreach, advertisements, and promotions such as presentations “on local news” or incentivizing PEHs “with a 10% discount card” at the VA cafeteria to encourage portal enrollment.

## Discussion

We conducted an exploratory qualitative study to characterize how PEHs enrolled in VA permanent supportive housing perceive and use MHV. We also identified barriers to and facilitators of sustained MHV engagement. Our findings underscore MHV's potential to enhance this population's healthcare access and autonomy while revealing inequities in portal adoption. Participants widely valued MHV for secure messaging, prescription management, and medical record access, functions that are critical for a population facing transportation barriers and fragmented care. At the same time, technical challenges [e.g., identity-verification (ID.me) and login complexity] and institutional gaps (e.g., limited proactive outreach, unclear points of contact for MHV support) hindered MHV adoption and ongoing use. Housing services within HUD-VASH appeared to support MHV utilization by enabling internet access and providing internet-capable devices, yet homelessness itself exacerbated digital exclusion due to device and internet insecurity.

Our findings are consistent with literature demonstrating that vulnerable populations, including persons with unstable housing, often lack reliable internet access, functional devices, or sufficient digital literacy to fully engage with patient portals (26). National survey data show that patient portal use has remained relatively low overall and disproportionately lower among people with lower income and other socially marginalized groups (27). These gaps may persist even among patients who are motivated to use digital tools to improve their health, suggesting that structural barriers such as internet access and digital literacy remain important (28). Participants’ descriptions of secure messaging, prescription management, review of laboratory and imaging results, and sharing records with non-VA providers suggest that MHV may support day-to-day healthcare management and self-guided care, particularly for patients managing chronic conditions.

Our findings highlight the need for equity-focused strategies that increase patient portal engagement among PEHs. Identity verification and multi-step log-ins may be especially burdensome for PEHs with limited device access, PTSD, cognitive or age-related impairments, and/or low digital literacy. Participants’ reports of confusion during account migration, authentication delays, technical errors, and frequent password resets suggest that the transition to more secure verification systems may have introduced additional barriers to sustained use for some PEHs. At the same time, MHV offers tools that are directly relevant to PEHs’ healthcare needs, including timely communication, medication continuity, and access to portable records. Improving equitable access to these functions may therefore help mitigate health disparities. This pattern is consistent with prior eHealth research suggesting that digital health tools can improve care engagement while also exacerbating inequities if barriers related to access and usability are not addressed (12).

Our findings directly identify several areas that may impede or facilitate patient portal engagement among PEHs, including portal usability, access to timely technical assistance, housing-related digital access, and support from fellow Veterans and VA staff. Building on these participant-reported experiences, several potential implementation strategies warrant further evaluation.

First, participants reported difficulty navigating MHV, completing authentication requirements, and obtaining timely technical assistance. These experiences highlight usability and support as important targets for improving patient portal engagement. Potential design adaptations, including simplified navigation for high-value tasks (e.g., refills, messaging, and appointments) could mitigate barriers for PEHs with PTSD, cognitive or age-related impairments, and/or low digital literacy. Our data also suggest that unclear support pathways and difficulty reaching timely technical assistance may reduce engagement, indicating that usability depends not only on interface design but also on whether users can quickly access human help when problems come up. Potential approaches include integrating plain-language prompts and a “human help” option earlier where security policy allows. These findings are consistent with prior VHA studies emphasizing the importance of organizational and workflow factors in shaping portal use. Fix et al. (29) found that although VA leadership promoted MHV, frontline clinicians and Veterans dealt with inconsistent role expectations and administrative burdens that limited effective engagement. For homeless-experienced Veterans in particular, login requirements for premium portal access, including multifactor authentication, may function as structural barriers due to unstable contact information, limited technology access, or disrupted documentation (30–32). Together, these findings highlight the importance of portal organization, flexible workflows, and clear points of contact to support portal engagement among disadvantaged Veterans, particularly within programs such as HUD-VASH.

Second, structured MHV supports at the time of HUD-VASH enrollment may improve MHV uptake and sustained engagement. Descriptions of more consistent portal use after obtaining stable housing suggest that housing context may shape the capacity to prioritize and sustain digital healthcare engagement over time alongside its ability to increase device and internet access. One potential approach arising from these findings is to provide structured MHV support during HUD-VASH enrollment or ongoing case management; however, the effectiveness and feasibility of such an approach were not evaluated in the present study. Potential approaches could include hands-on MHV onboarding, MHV workshops, brief print materials or walk-throughs of key portal functions, and ongoing technical support from HUD-VASH teams. During the COVID-19 pandemic, the VA's digital divide program (33)—which provides VA-loaned devices for telehealth, including a “white glove” service in which a staff member walks participants through steps needed for device usage—increased access to telehealth technology for vulnerable populations including PEHs. Although these devices are currently limited to virtual visits with healthcare providers, similar infrastructure could be leveraged or expanded to enable greater access to MHV and other digital health technologies (e.g., mobile applications for mental health disorders), particularly when paired with accessory technology such as mobile hotspots.

Finally, participants directly described fellow Veterans and VA peer-support staff as important sources of information and assistance with MHV, particularly when formal outreach was limited, inconsistent, or not consistently trusted. Participants also identified peer-led onboarding programs as a potential approach for supporting MHV use. Peer support specialists with lived expertise in homelessness and/or behavioral health diagnoses are already embedded within VA homeless programs and HUD-VASH teams, positioning them well to support MHV enrollment and use (34, 35). This finding builds on prior evidence that peer support and tailored outreach can facilitate digital health uptake among vulnerable populations. Garvin et al (32) found that peer-delivered digital literacy coaching increased telehealth use among homeless-experienced Veterans, in part by reducing anxiety and increasing trust through shared lived experience. Leveraging peer support specialists and the HUD-VASH workforce to assist with MHV use may offer an equity-oriented strategy for addressing barriers related to trust and access. Future research could evaluate whether formally incorporating peer support specialists or other HUD-VASH staff into MHV support improves portal adoption or sustained engagement. Collectively, these findings reinforce the value of patient-centered implementation approaches that integrate tailored support, trauma-informed design, and equitable access to digital health infrastructure.

Implementation of these strategies may be shaped by organizational resources and context. Potential barriers include staff capacity and competing workflow demands, the need to balance simplified access with security, and continued instability in device or internet access among populations experiencing housing instability. The VA context may also influence the feasibility of these approaches. As an integrated healthcare system, the VA has existing infrastructure (e.g., HUD-VASH teams, peer support specialists, digital access programs) that may not be available in other healthcare or homeless program settings. Barriers identified in this study, including authentication difficulties, limited digital literacy, inconsistent technology access, and unclear pathways to technical support may also be relevant to other populations experiencing housing instability. Future research should examine how strategies to address these barriers can be adapted and implemented across different healthcare and service settings.

### Limitations

This study has limitations. First, findings are from a small sample (*n* = 17) of PEHs at a single VA site (Greater Los Angeles), which may limit transferability to rural or non-VA populations. However, we note that this site has VA's largest homeless program (and largest HUD-VASH program) and is thus a reasonable place for initial data collection that can inform larger studies. Second, these findings would benefit from integration with clinical factors found in the electronic health record to be associated with MHV use. Finally, our use of telephone interviews reflected our study's resources but likely underrepresented more vulnerable PEHs without access to a phone line and/or with less stable housing.

## Conclusions

Identifying tailored strategies to support patient portal adoption among PEHs is critical to advancing health equity. Additional data is needed—from a larger national sample, with varying housing status and technology skills—to build upon this study. Future research could test implementation strategies (e.g., peer navigation programs and simplified MHV interfaces) for MHV within HUD-VASH and other homeless program settings to explore their impacts on portal adoption. Enhanced VA device subsidy programs and better integration of digital health technology within homeless services may be valuable to address the digital divide faced by PEHs that impacts their MHV use. These findings likely reflect a larger context of needing tailored, equity-focused strategies to address inequities in digital healthcare solutions for vulnerable populations, within and outside VA.

## Data Availability

The raw data supporting the conclusions of this article will be made available by the authors, without undue reservation.

## Footnotes

^†^Harris T, Panadero T, Chassman S, et al. Enhancing Patient Portal Adoption Among Veterans with Experiences of Homelessness. Unpublished. Submitted for publication. 2026
