# Supplementary material for: Homeless-experienced patients’ perspectives on improving patient portal Use
Source: Front Digit Health. 2026 Sep 8;8:1861993. doi: 10.3389/fdgth.2026.1861993 (PMC13593823; doi:10.3389/fdgth.2026.1861993)
Supplement: Supplementary file 1 [file Datasheet1.pdf]

PARTICIPANT DEMOGRAPHIC, HEALTHCARE ENGAGEMENT, AND DIGITAL ACCESS QUESTIONNAIRE

DEMOGRAPHICS

1. What is your gender?
2. What is your race/ethnicity?
3. What is your current marital status?
   - o Never Married
   - o Divorced or Separated
   - o Married
   - o Other
4. What is your current housing status?
   - o Currently housed
   - o Sheltered homelessness (temporary housing, hotel/motel, doubling up)
   - o Unsheltered homelessness (car, park, street, other place not meant for human habitation)
   - o Institutional housing (rehab, inpatient program)

HEALTHCARE ENGAGEMENT

5. During the past 12 months, how many times have you seen a doctor or nurse?
6. Do you have a regular doctor?
   - o Yes
   - o No
   - o Not sure
7. Do you take any routine medications?
   - o Yes
   - o No
   - o Not sure

DIGITAL ACCESS

8. Do you have a working cell phone with minutes?
   - o Yes
   - o No
9. Is your cell phone a smartphone or not, or are you not sure?
   - o Yes
   - o No
   - o Don't Know
10. In the past 30 days did you send/receive any text messages on your cell phone?
    - o Yes
    - o No
    - o Not sure
11. Do you have access to a computer?
    - o Yes
    - o No
12. Do you ever go online to access the internet?
    - o Yes
    - o No
